# Supplementary material for: Characterization of Volatile Substances in Pu-erh Tea (Raw Tea) at Different Storage Times
Source: Foods. 2025 Feb 28;14(5):840. doi: 10.3390/foods14050840 (PMC11898881; doi:10.3390/foods14050840)
Supplement: Supplementary file 1 [file foods-14-00840-s001.zip › foods-3459801-supplementary.pdf]

Table S1 General table of aroma substances

No.	Compound name	CAS	Group	P1	P2	P3	P4	P5	P6	P7	P8	P9	P10	P11	P12	P13	P14
M1	3-Cyclohexen-1-ol, 4-methyl-1-(1-methylethyl)-, (R)-	20126-76-5	alcohols	0.32	0.39	0.44	0.35	0.54	0.46	0.56	0.50	0.55	0.47	0.50	0.39	0.47	0.70
M2	Eucalyptol	470-82-6	alcohols	0.05	0.07	0.05	0.04	0.09	0.07	0.05	0.05	0.06	0.09	0.04	0.03	0.04	0.05
M3	1H-3a,7-Methanoazulen-5-ol, octahydro-3,8,8-trimethyl-6-methylene-	28231-03-0	alcohols	0.10	0.07	0.07	0.08	0.20	0.41	0.25	0.47	0.23	0.32	0.12	0.13	0.19	0.10
M4	Linalool	78-70-6	alcohols	2.46	2.28	2.50	3.17	3.04	2.88	2.84	2.54	2.61	2.41	2.59	2.19	2.18	2.50
M5	2-[(2R,5S)-5-ethenyl-5-methyltetrahydrofuran-2-yl]propan-2-ol	5989-33-3	alcohols	0.25	0.32	0.34	0.39	0.42	0.37	0.50	0.33	0.46	0.29	0.56	0.24	0.49	0.78
M6	1-Octen-3-ol	3391-86-4	alcohols	0.26	0.22	0.27	0.25	0.26	0.20	0.25	0.21	0.25	0.13	0.24	0.22	0.18	0.13
M7	Geraniol	106-24-1	alcohols	0.03	0.05	0.05	0.07	0.07	0.05	0.08	0.05	0.05	0.03	0.04	0.03	0.04	0.04
M8	1-Octanol	111-87-5	alcohols	0.11	0.06	0.06	0.09	0.07	0.05	0.08	0.06	0.06	0.02	0.06	0.13	0.11	0.03
M9	Bicyclo[2.2.1]heptan-2-ol,1,7,7-trimethyl-, (1S-endo)-	464-45-9	alcohols	0.04	0.04	0.04	0.05	0.07	0.07	0.06	0.05	0.06	0.06	0.06	0.04	0.06	0.07
M10	Fenchol, exo-	22627-95-8	alcohols	0.05	0.06	0.04	0.04	0.07	0.07	0.07	0.07	0.08	0.07	0.05	0.04	0.04	0.05
M11	trans-Linalool oxide (furanoid)	34995-77-2	alcohols	0.22	0.26	0.26	0.55	0.29	0.25	0.30	0.18	0.28	0.15	0.56	0.10	0.44	0.50
M12	Cyclohexanol,1-methyl-4-(1-methylethyl)-	21129-27-1	alcohols	0.02	0.02	0.01	0.02	0.02	0.04	0.02	0.02	0.03	0.01	0.01	0.01	0.01	0.01
M13	1,5,7-Octatrien-3-ol, 3,7-dimethyl-	29957-43-5	alcohols	0.44	0.55	-	0.58	0.44	0.56	-	0.48	0.47	0.39	0.30	0.28	0.40	0.39
M14	(3R,6S)-2,2,6-Trimethyl-6-vinyltetrahydro-2H-pyran-3-ol	39028-58-5	alcohols	0.03	0.03	0.03	0.04	0.04	-	0.03	-	0.03	0.02	0.05	-	0.04	0.06
M15	2-Nonen-1-ol	22104-79-6	alcohols	0.67	0.60	0.51	0.53	0.49	0.40	-	0.52	0.52	0.43	0.85	0.58	-	-
M16	2-Cyclohexen-1-ol, 1-methyl-4-(1-methylethyl)-, cis-	29803-82-5	alcohols	-	0.01	0.01	-	0.02	0.02	-	0.01	0.01	0.02	0.01	0.01	-	0.01
M17	1-Hexanol	111-27-3	alcohols	0.00	-	0.01	0.01	-	-	0.01	0.01	0.01	0.01	0.01	0.01	0.01	-
M18	Benzyl alcohol	100-51-6	alcohols	-	0.02	-	0.01	0.02	0.04	0.02	-	-	0.01	0.02	0.02	0.02	-
M19	3-Hexen-1-ol	544-12-7	alcohols	-	-	0.02	0.02	0.02	0.02	0.01	0.02	-	-	0.02	-	0.01	0.01
M20	L-α-Terpineol	10482-56-1	alcohols	0.97	-	1.04	1.02	1.41	-	1.45	-	-	-	-	1.17	1.36	1.91
M21	2-Decen-1-ol, (Z)-	4194-71-2	alcohols	0.30	-	-	-	-	0.10	-	0.14	0.14	-	0.34	0.24	-	-
M22	Benzenemethanol, α,α,4-trimethyl-	1197-01-9	alcohols	0.01	-	-	-	-	-	0.01	-	-	-	0.01	0.01	-	0.02
M23	2,6-Octadien-1-ol, 3,7-dimethyl-, (Z)-	106-25-2	alcohols	-	-	-	-	0.01	-	-	0.19	-	-	-	-	0.02	0.03
M24	2-Cyclohexen-1-ol, 2,4,4-trimethyl-	73741-61-4	alcohols	0.06	-	-	-	-	-	-	-	0.07	-	-	0.06	0.04	-
M25	2H-Pyran-3-ol, 6-ethenyltetrahydro-2,2,6-trimethyl-	14049-11-7	alcohols	-	-	-	-	-	0.03	-	-	0.02	-	-	0.02	-	-
M26	Cyclopentanol, 2-methyl-	24070-77-7	alcohols	-	-	1.02	-	-	-	-	-	-	-	1.12	0.71	-	-
M27	(3R,3aR,5S,6R,7aR)-3,6,7,7-Tetramethyloctahydro-3a,6-ethanoinden-5-ol	50657-30-2	alcohols	-	-	-	-	-	0.01	-	-	0.02	0.01	-	-	-	-
M28	1-Heptanol	111-70-6	alcohols	-	-	0.01	-	-	-	-	0.01	-	-	-	-	-	-
M29	3-Pentanol, 2-methyl-	565-67-3	alcohols	-	-	0.00	0.00	-	-	-	-	-	-	-	-	-	-
M30	Tau-Cadinol acetate	149197-48-8	alcohols	-	-	-	-	-	-	-	-	0.01	-	-	-	-	-
M31	2-Octen-1-ol, (Z)-	26001-58-1	alcohols	-	-	0.07	-	-	-	-	-	-	-	-	-	-	-
M32	Cyclohexanol, 2-methyl-5-(1-methylethenyl)-	619-01-2	alcohols	-	-	-	-	-	0.01	-	-	-	-	-	-	-	-
M33	Bicyclo[2.2.1]heptan-2-ol, 2,3,3-trimethyl-	465-31-6	alcohols	0.09	0.08	0.09	0.08	0.14	0.12	0.11	0.12	-	0.11	-	0.07	0.09	-
M34	(1S,3S,4S,5R)-1-Isopropyl-4-methylbicyclo[3.1.0]hexan-3-ol	7712-79-0	alcohols	-	-	-	-	0.01	-	-	-	-	-	-	-	-	-
M35	Phenol, 4-ethyl-2-methoxy-	2785-89-9	Phenols	-	0.01	0.01	-	0.02	0.01	0.02	-	0.05	-	0.01	-	0.01	0.03
M36	Phenol, 2-methyl-	95-48-7	Phenols	-	-	0.01	-	0.02	0.01	0.16	-	0.03	-	0.01	-	0.01	0.03
M37	Phenol	108-95-2	Phenols	-	0.01	0.01	-	0.02	0.02	0.01	-	0.02	-	-	0.01	-	-
M38	Phenol, 3-methyl-	108-39-4	Phenols	-	-	-	-	-	0.03	-	-	-	0.05	-	-	-	-
M39	2,5-Diethylphenol	876-20-0	Phenols	-	-	-	-	-	-	-	-	-	-	-	-	-	0.01
M40	Creosol	93-51-6	Phenols	-	-	-	-	-	-	-	-	-	0.12	-	-	-	-
M41	Phenol, 2,3,6-trimethyl-	2416-94-6	Phenols	-	-	-	-	-	-	-	-	-	-	-	-	-	0.01
M42	Benzyl nitrile	3682-17-5	Acids	0.01	0.01	0.02	-	-	-	-	-	-	-	-	-	-	-
M43	2-Nonenal, (E)-	18829-56-6	Aldehydes	0.04	0.03	0.03	0.04	0.03	0.05	0.04	0.03	0.07	0.03	0.08	0.03	0.03	0.07
M44	(E,E)-2,4-Heptadienal	4313-03-5	Aldehydes	0.66	0.59	0.68	0.61	0.86	0.74	0.39	0.66	0.88	0.67	0.91	0.62	0.82	1.05
M45	2-Heptenal, (Z)-	57266-86-1	Aldehydes	0.07	0.07	0.05	0.04	0.05	0.05	0.06	0.04	0.08	0.03	0.13	0.05	0.06	0.05

M46	β-Cyclocitral	432-25-7	Aldehydes	0.28	0.19	0.22	0.22	0.26	0.21	0.28	0.27	0.32	0.16	0.30	0.26	0.17	0.26
M47	Benzaldehyde	100-52-7	Aldehydes	0.81	0.76	0.85	0.76	0.95	0.94	1.16	1.16	1.30	1.08	1.10	1.04	0.91	1.38
M48	Benzeneacetaldehyde	122-78-1	Aldehydes	0.16	0.16	0.18	0.22	0.21	0.22	0.32	0.27	0.25	0.25	0.28	0.26	0.21	0.32
M49	1,3-Cyclohexadiene-1-carboxaldehyde, 2,6,6-trimethyl-	116-26-7	Aldehydes	0.43	0.35	0.34	0.39	0.37	0.34	0.39	0.36	0.45	0.27	0.40	0.36	0.29	0.37
M50	2-Octenal, (E)-	2548-87-0	Aldehydes	0.15	0.12	0.14	0.11	0.15	0.15	0.16	0.13	0.21	0.10	0.20	0.08	0.13	0.20
M51	Heptanal	111-71-7	Aldehydes	0.36	0.29	0.34	0.31	0.35	0.37	0.33	0.29	0.39	0.24	0.37	0.23	0.27	0.38
M52	2-Pentenal, (E)-	1576-87-0	Aldehydes	0.10	0.09	0.12	0.10	0.13	0.13	0.11	0.13	0.16	0.10	0.16	0.10	0.16	0.15
M53	3-Cyclohexene-1-acetaldehyde, α,4-dimethyl-	29548-14-9	Aldehydes	0.03	0.03	0.04	0.04	0.05	0.04	0.04	0.04	0.05	0.03	0.04	0.03	0.02	0.05
M54	2-Hexenal	505-57-7	Aldehydes	0.20	0.16	0.20	0.25	0.22	0.24	0.20	0.14	0.26	0.13	0.30	0.10	0.29	0.36
M55	4-Heptenal	62238-34-0	Aldehydes	0.22	0.15	0.16	0.15	0.19	0.18	0.17	0.17	0.23	0.14	-	0.12	0.14	0.20
M56	Pentanal, 2-methyl-	123-15-9	Aldehydes	0.02	0.01	0.02	0.01	-	0.02	0.01	0.01	0.01	0.01	0.01	0.01	0.01	-
M57	Octanal	124-13-0	Aldehydes	0.29	0.28	0.27	0.25	0.23	0.23	-	0.22	0.29	-	0.25	0.20	0.19	-
M58	α-Cyclocitral	432-24-6	Aldehydes	0.06	-	-	0.03	0.02	-	0.05	0.06	0.06	0.02	0.06	0.05	0.03	0.03
M59	β-Homocyclocitral	472-66-2	Aldehydes	0.01	0.01	0.01	0.01	0.01	0.01	0.01	0.01	0.01	-	-	0.01	-	-
M60	Undecanal	112-44-7	Aldehydes	0.01	0.03	0.02	0.02	-	-	-	-	-	0.01	0.01	0.01	0.01	0.01
M61	4-Heptenal, (Z)-	6728-31-0	Aldehydes	0.02	0.13	0.17	-	0.20	-	-	0.16	0.01	-	0.19	-	0.14	0.22
M62	Decanal	112-31-2	Aldehydes	-	0.08	0.29	0.42	0.06	-	-	-	0.18	0.19	-	-	0.24	0.17
M63	Dodecanal	112-54-9	Aldehydes	0.01	0.01	0.00	0.01	-	-	-	-	-	0.00	0.00	0.01	0.01	-
M64	Benzaldehyde, 4-ethyl-	4748-78-1	Aldehydes	-	0.01	0.01	-	0.04	0.02	-	-	0.02	-	-	0.02	-	0.03
M65	Hexanal	66-25-1	Aldehydes	-	0.82	-	0.87	1.16	0.01	0.85	-	-	0.82	0.03	-	-	-
M66	Benzaldehyde, 2,4-dimethyl-	15764-16-6	Aldehydes	0.02	-	-	0.03	-	-	0.01	-	0.02	-	-	-	0.02	-
M67	3-Cyclohexene-1-carboxaldehyde, 4-methyl-	7560-64-7	Aldehydes	0.02	-	-	-	-	0.02	0.01	-	-	0.02	-	-	-	-
M68	Benzaldehyde, 2,4,6-trimethyl-	487-68-3	Aldehydes	-	-	-	-	-	-	-	-	0.02	0.03	0.02	-	-	-
M69	2,4-Nonadienal, (E,E)-	5910-87-2	Aldehydes	-	-	-	-	-	-	-	-	-	0.01	0.01	-	-	-
M70	Furfural	98-01-1	Aldehydes	-	0.01	0.01	-	-	-	-	-	-	-	-	-	-	-
M71	Citronellal	106-23-0	Aldehydes	-	-	0.02	-	-	-	-	-	-	-	-	-	-	-
M72	Benzaldehyde, 2-ethyl-	22927-13-5	Aldehydes	-	-	-	-	-	-	-	-	-	-	0.02	-	-	-
M73	2-Butenal, 2-methyl-, (E)-	497-03-0	Aldehydes	-	-	-	-	-	-	0.02	-	-	-	-	-	-	-
M74	Benzaldehyde, 4-methyl-	104-87-0	Aldehydes	-	-	-	-	-	-	-	-	-	-	-	-	0.01	-
M75	3,5-Octadien-2-one, (E,E)-	30086-02-3	Ketones	0.12	0.10	0.11	0.12	0.12	0.15	0.15	0.13	0.14	0.13	0.14	0.12	0.13	0.21
M76	2,2,6-trimethyl-Cyclohexanone	2408-37-9	Ketones	0.54	0.38	0.44	0.48	0.43	0.37	0.51	0.58	0.62	0.32	0.58	0.51	0.34	0.50
M77	2-Heptanone	110-43-0	Ketones	0.17	0.16	0.20	0.17	0.19	0.17	0.20	0.20	0.27	0.15	0.22	0.16	0.17	0.22
M78	2-Decanone	693-54-9	Ketones	0.04	0.05	0.04	0.04	0.05	0.04	0.05	0.04	0.05	0.02	0.03	0.03	0.02	0.02
M79	2-Octanone	111-13-7	Ketones	0.04	0.05	0.05	0.04	0.04	0.03	0.04	0.04	0.07	0.02	0.03	0.05	0.02	0.03
M80	3,5-Octadien-2-one	38284-27-4	Ketones	0.08	0.10	0.10	0.09	0.13	0.10	0.12	0.10	0.11	0.09	0.12	0.11	0.11	0.14
M81	2-Cyclohexen-1-one, 3-methyl-	1193-18-6	Ketones	0.23	0.18	0.20	0.18	0.20	0.17	0.23	0.25	0.29	0.17	0.26	0.22	0.19	0.26
M82	3-Octanone	106-68-3	Ketones	0.03	0.03	0.04	0.04	0.05	0.04	0.05	0.05	0.06	0.05	0.06	0.03	0.03	0.04
M83	4-(2,6,6-Trimethylcyclohexa-1,3-dienyl)but-3-en-2-one	1203-08-3	Ketones	0.01	0.02	0.02	0.02	0.03	0.03	0.03	0.03	0.02	0.03	0.02	0.03	0.02	0.03
M84	(R,S)-5-Ethyl-6-methyl-3E-hepten-2-one	57283-79-1	Ketones	0.05	0.04	0.05	0.05	0.04	0.05	0.06	0.05	0.09	0.03	0.06	0.03	0.04	0.05
M85	α-Ionone	127-41-3	Ketones	0.06	0.04	0.05	0.05	0.06	0.06	0.07	0.06	0.06	0.06	0.07	0.07	0.05	0.10
M86	3-Buten-2-one, 4-(2,6,6-trimethyl-1-cyclohexen-1-yl)-	14901-07-6	Ketones	0.14	0.14	0.14	0.13	0.18	0.17	0.21	0.20	0.15	0.20	0.20	0.19	0.15	0.26
M87	Acetophenone	98-86-2	Ketones	0.11	0.11	0.10	0.11	0.15	0.12	0.13	0.11	0.15	0.13	0.13	0.16	0.12	0.18
M88	(E)-Damascenone	23726-93-4	Ketones	0.02	0.02	0.02	0.03	0.03	0.03	0.03	0.03	0.03	0.02	0.03	0.02	0.02	0.02
M89	5-Hepten-2-one, 6-methyl-	110-93-0	Ketones	0.37	0.37	0.40	0.37	0.41	0.38	0.38	0.36	0.46	0.30	0.38	0.35	0.28	0.34
M90	5,9-Undecadien-2-one, 6,10-dimethyl-, (E)-	3796-70-1	Ketones	0.03	0.03	0.04	0.04	0.05	0.04	0.05	0.05	0.04	0.03	0.04	0.04	0.04	0.07
M91	2-Hexanone	591-78-6	Ketones	0.01	0.01	0.02	0.01	0.03	0.01	0.02	0.02	0.02	0.01	0.02	0.01	0.02	0.02
M92	2-Undecanone	112-12-9	Ketones	0.01	0.01	0.01	0.01	0.01	0.01	0.01	0.01	0.01	0.01	0.01	0.01	0.00	0.01
M93	Bicyclo[2.2.1]heptan-2-one, 1,7,7-trimethyl-, (1S)-	464-48-2	Ketones	0.03	0.02	0.03	0.03	0.05	0.08	0.03	0.08	0.07	0.06	0.04	0.03	0.04	-
M94	2-Undecanone, 6,10-dimethyl-	1604-34-8	Ketones	0.01	-	0.01	0.01	0.01	0.01	0.01	0.01	0.01	0.01	0.01	-	0.01	0.01
M95	2,6,6-Trimethyl-2-cyclohexene-1,4-dione	1125-21-9	Ketones	-	-	0.01	0.01	-	0.01	0.01	0.01	-	0.01	0.02	0.01	0.02	0.01
M96	Isophorone	78-59-1	Ketones	-	0.01	-	0.01	-	0.01	-	-	-	0.01	0.01	-	0.01	0.03

M97	2-Butanone, 4-(2,6,6-trimethyl-1-cyclohexen-1-yl)-	17283-81-7	Ketones	-	-	-	-	-	-	0.01	0.02	-	-	0.01	0.01	-	0.02
M98	2-Pentadecanone, 6,10,14-trimethyl-	502-69-2	Ketones	-	-	-	-	-	-	0.02	0.00	-	-	0.01	0.01	-	0.01
M99	2-Cyclopenten-1-one, 2,3-dimethyl-	1121-05-7	Ketones	-	-	-	-	-	-	-	0.01	-	0.02	0.01	-	-	0.03
M100	3-Penten-2-one, 4-methyl-	141-79-7	Ketones	-	-	0.16	0.18	-	-	-	0.15	-	-	-	0.16	-	-
M101	3-Pentanone, 2-methyl-	565-69-5	Ketones	-	-	-	-	-	-	-	0.02	-	0.03	-	0.02	-	0.02
M102	2-Nonanone	821-55-6	Ketones	-	-	0.07	-	0.09	0.06	-	0.08	-	-	-	-	-	-
M103	2H-Pyran-3(4H)-one, 6-ethenyldihydro-2,2,6-trimethyl-	33933-72-1	Ketones	-	-	-	-	-	-	0.07	0.07	-	-	-	-	0.06	-
M104	1H-Inden-1-one, 2,3-dihydro-	83-33-0	Ketones	-	-	-	-	-	-	-	-	-	0.01	-	-	-	0.03
M105	Cyclohexanone	108-94-1	Ketones	-	-	-	-	-	0.01	-	-	-	-	-	-	-	-
M106	3,5-Heptadien-2-one, 6-methyl-, (E)-	16647-04-4	Ketones	0.06	-	-	-	-	-	-	-	-	-	-	-	-	-
M107	3-Octen-2-one, (E)-	18402-82-9	Ketones	-	-	-	-	-	-	-	-	-	-	-	-	0.02	-
M108	Ethanone, 1-(3-methylphenyl)-	585-74-0	Ketones	-	-	-	-	-	-	-	-	-	0.02	-	-	-	-
M109	2,5-Cyclohexadiene-1,4-dione,2,6-bis(1,1-dimethylethyl)-	719-22-2	Ketones	0.01	0.01	0.01	0.01	0.01	0.01	0.01	0.01	0.01	0.02	0.01	0.01	0.02	0.02
M110	2-Heptanone, 6-methyl-	928-68-7	Ketones	-	0.02	-	-	-	-	-	-	-	-	-	-	-	-
M111	Methyl salicylate	119-36-8	Esters	0.15	0.12	0.17	0.32	0.21	0.15	0.18	0.12	0.10	0.10	0.26	0.07	0.14	0.08
M112	Hexanoic acid, ethyl ester	123-66-0	Esters	0.13	0.07	0.10	0.08	0.08	0.10	0.12	0.05	0.10	0.04	0.11	0.01	0.03	0.02
M113	Benzoic acid, methyl ester	93-58-3	Esters	0.02	0.02	0.01	0.01	0.02	0.02	0.02	0.02	0.02	0.02	0.02	0.01	0.02	0.02
M114	2(4H)-Benzofuranone, 5,6,7,7a-tetrahydro-4,4,7a-trimethyl-	15356-74-8	Esters	0.03	0.04	0.02	0.04	0.03	0.04	0.05	0.06	0.03	0.05	0.05	0.05	0.05	0.06
M115	Acetic acid, phenylmethyl ester	140-11-4	Esters	0.05	0.02	0.03	0.05	0.07	0.05	0.04	0.05	0.06	0.04	0.06	0.04	0.04	0.08
M116	(+)-Borneol acetate	20347-65-3	Esters	-	0.01	-	0.01	0.09	0.01	0.02	0.02	0.02	0.02	0.01	-	0.00	-
M117	Octanoic acid, ethyl ester	106-32-1	Esters	0.04	0.03	0.05	0.04	0.05	0.05	0.04	-	0.03	-	-	-	-	-
M118	Nonanoic acid, ethyl ester	123-29-5	Esters	-	0.00	0.01	0.01	0.01	-	-	0.01	-	-	-	-	-	-
M119	Hexanoic acid, 3-hexenyl ester, (Z)-	31501-11-8	Esters	0.01	0.01	0.01	0.04	0.01	-	-	-	-	-	-	-	-	-
M120	Hexanoic acid, hexyl ester	6378-65-0	Esters	0.02	-	-	0.01	-	-	-	0.01	-	-	0.00	-	-	-
M121	Butanoic acid, 2-methyl-, hexyl ester	10032-15-2	Esters	-	0.01	0.01	0.01	-	-	-	-	-	-	-	-	-	-
M122	Butanoic acid, 3-hexenyl ester, (Z)-	16491-36-4	Esters	-	-	0.01	0.03	-	-	-	-	-	-	0.01	-	-	-
M123	Propanoic acid, 2-methyl-, 3-hydroxy-2,2,4-trimethylpentyl ester	77-68-9	Esters	-	0.01	-	-	-	0.00	0.01	-	-	-	-	-	-	-
M124	Nonanoic acid, methyl ester	1731-84-6	Esters	-	-	-	-	0.01	0.01	-	-	-	-	-	-	-	-
M125	Butanoic acid, 3-hexenyl ester, (E)-	53398-84-8	Esters	0.01	0.00	-	-	-	-	-	-	-	-	-	-	-	-
M126	Benzeneacetic acid, methyl ester	101-41-7	Esters	-	-	-	-	-	-	-	-	0.01	-	-	-	-	-
M127	Hexanoic acid, methyl ester	106-70-7	Esters	-	-	-	-	-	-	-	-	-	-	0.02	-	-	-
M128	2(4H)-Benzofuranone, 5,6,7,7a-tetrahydro-4,4,7a-trimethyl-, (R)-	17092-92-1	Esters	0.03	-	-	-	-	-	-	-	-	-	-	-	-	-
M129	Methyl 6,6-dimethylbicyclo[3.1.1]hept-2-ene-2-carboxylate	30649-97-9	Esters	-	-	-	-	-	-	0.01	-	-	-	-	-	-	-
M130	cis-3-Hexenyl-α-methylbutyrate	53398-85-9	Esters	-	-	-	0.02	-	-	-	-	-	-	-	-	-	-
M131	Hexanoic acid, 2-hexenyl ester, (E)-	53398-86-0	Esters	-	-	-	0.01	-	-	-	-	-	-	-	-	-	-
M132	Hexanoic acid, butyl ester	626-82-4	Esters	-	-	0.01	-	-	-	-	-	-	-	-	-	-	-
M133	2,2,4-Trimethyl-1,3-pentanediol diisobutyrate	6846-50-0	Esters	-	-	-	-	-	-	0.04	-	-	-	-	-	-	-
M134	α-Terpinyl acetate	80-26-2	Esters	-	-	-	-	-	-	-	-	0.01	-	-	-	-	-
M135	Benzene, 1,2,4-trimethyl-	95-63-6	Aromatic hydrocarbons	0.12	0.10	0.09	0.09	0.09	0.09	0.11	0.10	0.14	0.06	0.11	0.07	0.06	0.11
M136	Naphthalene, 2-methyl-	91-57-6	Aromatic hydrocarbons	0.03	0.03	0.04	0.03	0.06	0.07	0.08	0.05	0.09	0.05	0.04	0.03	0.03	0.07
M137	Benzene, 1-methyl-4-(1-methylethenyl)-	1195-32-0	Aromatic hydrocarbons	0.34	0.34	0.35	0.34	0.41	0.39	0.46	0.44	0.57	0.25	0.41	0.26	0.19	0.36
M138	Styrene	100-42-5	Aromatic hydrocarbons	0.07	0.05	0.05	0.07	0.09	0.08	0.08	0.07	0.09	0.07	0.08	0.07	0.05	0.08
M139	Anthracene	120-12-7	Aromatic hydrocarbons	0.00	0.01	0.01	0.01	0.03	0.01	0.02	0.01	0.02	0.01	0.01	0.01	0.01	0.03

M140	Toluene	108-88-3	Aromatic hydrocarbons	0.35	0.28	0.37	0.33	0.41	0.36	0.41	0.41	0.46	0.30	0.38	0.41	0.26	0.34
M141	Biphenyl	92-52-4	Aromatic hydrocarbons	0.01	0.01	0.02	0.01	0.03	0.02	0.03	0.02	0.03	0.01	0.02	0.01	0.02	0.04
M142	o-Xylene	95-47-6	Aromatic hydrocarbons	0.07	0.05	0.07	0.07	0.09	0.08	0.09	0.08	0.10	0.07	0.10	0.07	0.05	0.10
M143	o-Cymene	527-84-4	Aromatic hydrocarbons	0.99	0.93	1.00	0.82	1.18	0.99	1.25	1.25	1.54	0.73	1.00	0.70	0.53	0.95
M144	Naphthalene	91-20-3	Aromatic hydrocarbons	0.17	0.20	0.21	0.18	0.24	0.26	0.25	0.21	0.30	0.21	0.21	0.17	0.16	0.25
M145	Naphthalene, 1,2,3-trimethyl-4-propenyl-, (E)-	26137-53-1	Aromatic hydrocarbons	0.01	0.03	0.03	0.04	0.06	0.16	0.09	0.04	0.05	0.13	0.02	0.07	0.20	0.10
M146	1,1'-Biphenyl, 3,4-diethyl-	61141-66-0	Aromatic hydrocarbons	0.01	0.02	0.04	0.03	0.06	0.03	0.02	0.03	0.01	0.02	0.01	0.02	0.02	0.02
M147	1,1'-Biphenyl,2,2',5,5'-tetramethyl-	3075-84-1	Aromatic hydrocarbons	0.03	0.01	0.01	0.02	0.03	0.04	0.03	0.02	0.02	0.05	0.03	0.02	0.07	0.02
M148	1H-Indene, 2,3-dihydro-1,1,4,5-tetramethyl-	16204-57-2	Aromatic hydrocarbons	0.01	0.01	0.01	0.01	0.01	0.01	0.01	0.01	0.01	0.01	0.01	0.01	0.01	0.01
M149	1H-Indene, 1-ethylidene-	2471-83-2	Aromatic hydrocarbons	0.03	0.03	0.02	0.03	0.05	0.05	0.05	0.03	0.07	0.05	0.03	0.02	0.03	0.06
M150	Benzene, 1,2,3-trimethyl-	526-73-8	Aromatic hydrocarbons	0.12	0.10	0.11	0.10	0.12	0.10	0.12	0.15	0.22	0.08	0.14	0.11	0.06	0.13
M151	Benzene, 1-ethyl-3-methyl-	620-14-4	Aromatic hydrocarbons	0.05	0.02	0.03	0.03	0.05	0.03	0.03	0.03	0.05	0.02	-	0.02	0.01	0.02
M152	Naphthalene, 1,2-dihydro-1,5,8-trimethyl-	4506-36-9	Aromatic hydrocarbons	0.05	0.05	0.05	0.04	0.06	0.06	0.07	0.06	0.08	-	0.07	0.05	0.04	0.09
M153	Naphthalene, 1,6-dimethyl-	575-43-9	Aromatic hydrocarbons	0.02	0.02	0.02	0.03	0.04	0.04	0.04	0.03	0.05	-	0.03	0.03	0.02	0.08
M154	Naphthalene, 1,4,6-trimethyl-	2131-42-2	Aromatic hydrocarbons	-	-	0.00	0.00	0.01	0.01	0.01	0.00	-	0.00	0.00	0.01	0.00	0.01
M155	Naphthalene, 2,7-dimethyl-	582-16-1	Aromatic hydrocarbons	0.01	0.01	0.01	0.01	0.01	0.01	0.01	0.01	-	0.01	0.02	-	-	-
M156	Benzene, 1,3-dimethyl-	108-38-3	Aromatic hydrocarbons	-	0.04	0.12	0.14	0.18	-	0.16	0.13	-	0.11	0.16	0.13	-	0.13
M157	p-Xylene	106-42-3	Aromatic hydrocarbons	0.14	0.10	0.04	-	0.07	0.14	-	-	0.17	-	0.06	0.08	0.10	-
M158	Naphthalene, 1,3-dimethyl-	575-41-7	Aromatic hydrocarbons	0.01	0.01	-	-	-	0.02	0.01	0.01	0.02	0.01	-	0.01	0.01	-
M159	1,1'-Biphenyl, 2-ethyl-	1812-51-7	Aromatic hydrocarbons	-	0.01	0.01	-	0.01	0.05	0.02	-	-	0.04	-	0.02	-	0.02
M160	Benzene, 1-ethyl-2-methyl-	611-14-3	Aromatic hydrocarbons	-	-	-	0.01	0.02	0.01	0.02	0.01	0.01	0.01	0.03	-	-	-
M161	Naphthalene, 2,3,6-trimethyl-	829-26-5	Aromatic hydrocarbons	-	0.00	-	0.01	0.01	0.01	0.01	-	0.01	-	-	-	-	0.01
M162	2,2'-Dimethylbiphenyl	605-39-0	Aromatic hydrocarbons	-	-	-	0.00	0.01	0.02	0.01	-	-	0.01	-	0.01	-	0.01
M163	Naphthalene, 1,6,7-trimethyl-	2245-38-7	Aromatic hydrocarbons	-	-	-	-	-	0.01	0.01	-	0.01	-	0.01	0.01	0.01	0.03
M164	Acenaphthene	83-32-9	Aromatic hydrocarbons	-	-	-	0.00	-	0.01	0.00	-	0.01	0.01	-	-	0.00	0.03
M165	Naphthalene, 1,2-dimethyl-	573-98-8	Aromatic hydrocarbons	-	-	-	-	-	0.01	-	0.01	-	0.01	-	-	-	0.05
M166	Benzene, 1,1'-ethylidenebis-	612-00-0	Aromatic hydrocarbons	-	-	-	0.01	-	-	-	-	0.01	0.01	0.01	-	-	-
M167	(3R,4aS,5R)-4a,5-Dimethyl-3-(prop-1-en-2-yl)-1,2,3,4,4a,5,6,7-octahydronaphthalene	24741-64-8	Aromatic hydrocarbons	-	-	-	0.01	-	0.01	-	-	-	-	-	-	-	0.01
M168	α-Calacorene	21391-99-1	Aromatic	-	-	-	-	-	0.01	-	0.01	-	-	-	-	-	-

			hydrocarbons														
M169	Naphthalene, 1,2-dihydro-1,1,6-trimethyl-	30364-38-6	Aromatic hydrocarbons	-	0.04	-	-	-	-	0.06	-	-	-	-	-	-	-
M170	Naphthalene, 1,2,4a,5,6,8a-hexahydro-4,7-dimethyl-1-(1-methylethyl)-, (1α,4aα,8aα)-	31983-22-9	Aromatic hydrocarbons	-	0.00	0.00	-	-	-	-	-	-	-	-	-	-	-
M171	Naphthalene, 1,4-dimethyl-	571-58-4	Aromatic hydrocarbons	0.02	-	-	-	-	-	0.03	-	-	-	-	-	-	-
M172	1,1'-Biphenyl, 2,4'-dimethyl-	611-61-0	Aromatic hydrocarbons	-	-	-	-	-	0.02	0.01	-	-	-	-	-	-	-
M173	1,1'-Biphenyl, 4-methyl-	644-08-6	Aromatic hydrocarbons	-	-	-	-	-	-	-	-	-	0.01	-	-	-	0.03
M174	Benzene, 1-ethyl-2,4-dimethyl-	874-41-9	Aromatic hydrocarbons	0.02	-	-	-	-	-	0.01	-	-	-	-	-	-	-
M175	Naphthalene, 1-ethyl-	1127-76-0	Aromatic hydrocarbons	-	-	-	-	-	0.01	-	-	-	-	-	-	-	-
M176	(1R,4aS,8aR)-1-Isopropyl-4,7-dimethyl-1,2,4a,5,6,8a-hexahydronaphthalene	20085-19-2	Aromatic hydrocarbons	-	-	-	-	-	-	-	-	-	0.01	-	-	-	-
M177	Benzene, 2-ethyl-1,3-dimethyl-	2870-04-4	Aromatic hydrocarbons	-	-	-	-	-	0.01	-	-	-	-	-	-	-	-
M178	Benzene, pentyl-	538-68-1	Aromatic hydrocarbons	-	-	-	-	-	-	-	-	-	-	-	0.01	-	-
M179	Naphthalene, 1,5-dimethyl-	571-61-9	Aromatic hydrocarbons	-	-	-	-	0.01	-	-	-	-	-	-	-	-	-
M180	Naphthalene, 1,7-dimethyl-	575-37-1	Aromatic hydrocarbons	-	-	-	0.01	-	-	-	-	-	-	-	-	-	-
M181	Naphthalene, 2,3-dimethyl-	581-40-8	Aromatic hydrocarbons	-	-	-	-	-	-	-	-	-	0.01	-	-	-	-
M182	(1S,4aR,8aS)-1-Isopropyl-7-methyl-4-methylene-1,2,3,4,4a,5,6,8a-octahydronaphthalene	6980-46-7	Aromatic hydrocarbons	-	-	-	-	-	-	-	-	0.01	-	-	-	-	-
M183	trans-Calamenene	73209-42-4	Aromatic hydrocarbons	-	-	0.01	-	-	-	-	-	-	-	-	-	-	-
M184	Naphthalene, 1-methyl-	90-12-0	Aromatic hydrocarbons	-	-	-	0.03	-	-	-	-	-	-	-	-	-	-
M185	Benzene, 4-ethyl-1,2-dimethyl-	934-80-5	Aromatic hydrocarbons	-	-	-	-	-	0.01	-	-	-	-	-	-	-	-
M186	1H-Indene, 2,3-dihydro-1,1,4,6-tetramethyl-	941-60-6	Aromatic hydrocarbons	-	-	-	-	-	0.01	-	-	-	-	-	-	-	-
M187	Fluorene	86-73-7	Aromatic hydrocarbons	-	0.02	0.02	0.02	0.04	0.02	0.02	-	0.03	0.02	0.02	0.03	-	0.08
M188	Mesitylene	108-67-8	Aromatic hydrocarbons	-	-	-	-	-	-	-	-	-	-	0.01	-	-	-
M189	Ethylbenzene	100-41-4	Aromatic hydrocarbons	0.03	0.02	-	-	-	0.04	0.05	-	0.05	0.06	-	-	-	-
M190	(1R,2S,6S,7S,8S)-8-Isopropyl-1-methyl-3-methylenetricyclo[4.4.0.02,7]decane-rel-	18252-44-3	Alkanes	0.01	-	-	-	-	0.03	0.02	0.04	0.02	0.03	-	0.01	0.01	0.01
M191	Bicyclo[2.2.1]heptane, 7,7-dimethyl-2-methylene-	471-84-1	Alkanes	-	0.21	-	0.21	-	0.24	0.18	-	-	-	-	-	-	-
M192	Pentadecane, 2,6,10,14-tetramethyl-	1921-70-6	Alkanes	-	-	0.00	-	-	-	-	-	-	-	-	0.00	-	-
M193	Heptane, 2,4-dimethyl-	2213-23-2	Alkanes	0.03	-	-	-	-	-	-	-	-	-	-	0.02	-	-
M194	Pentadecane, 2-methyl-	1560-93-6	Alkanes	-	-	-	-	-	-	0.01	-	-	-	-	-	-	-
M195	Hexane, 2,2,5-trimethyl-	3522-94-9	Alkanes	-	-	-	-	-	-	-	-	-	-	0.03	-	-	-
M196	2-Hexene, 3,5,5-trimethyl-	26456-76-8	Olefins	0.04	0.05	0.05	0.05	0.06	0.04	0.05	0.04	0.05	0.03	0.05	0.04	0.05	0.03
M197	trans-β-Ocimene	3779-61-1	Olefins	0.22	0.23	0.28	0.24	0.28	0.26	0.28	0.25	0.33	0.27	0.17	0.13	0.09	0.12
M198	1,3-Cyclopentadiene, 1,2,5,5-tetramethyl-	4249-12-1	Olefins	0.21	0.19	0.21	0.21	0.23	0.19	0.23	0.22	0.23	0.15	0.18	0.13	0.06	0.14
M199	1,3-cis,5-cis-Octatriene	40087-62-5	Olefins	0.02	0.02	0.02	0.02	0.03	0.02	0.02	0.01	0.02	0.01	0.01	0.01	0.01	0.01

M200	2,4,6-Octatriene, 2,6-dimethyl-, (E,E)-	3016-19-1	Olefins	0.02	0.02	0.02	0.02	0.02	0.02	0.02	0.03	0.03	0.01	0.02	0.01	0.02	0.01
M201	α-Phellandrene	99-83-2	Olefins	0.23	0.17	0.20	0.13	0.20	0.17	0.19	0.19	0.20	0.10	0.12	0.10	0.06	0.09
M202	α-Terpinene	99-86-5	Olefins	0.36	0.39	0.45	0.38	0.48	0.42	0.49	0.46	0.49	0.31	0.33	0.25	0.13	0.24
M203	γ-Terpinene	99-85-4	Olefins	0.42	0.35	0.42	0.37	0.44	0.40	0.50	0.44	0.45	0.28	0.32	0.27	0.13	0.23
M204	Cedrene	11028-42-5	Olefins	0.02	0.02	0.02	0.02	0.03	0.04	0.02	0.05	0.03	0.05	0.03	0.02	0.02	0.02
M205	2,4,6-Octatriene, 2,6-dimethyl-, (E,Z)-	7216-56-0	Olefins	0.03	0.03	0.03	0.03	0.04	0.03	0.04	0.04	0.04	0.02	0.02	0.02	0.01	0.02
M206	1-methyl-4-(1-methylethylidene)-Cyclohexene	586-62-9	Olefins	0.56	0.59	0.62	0.50	0.62	0.56	0.62	0.65	0.72	0.42	0.46	0.38	0.20	0.36
M207	β-Myrcene	123-35-3	Olefins	0.28	0.29	0.34	0.31	0.41	0.30	0.37	0.34	0.32	0.20	0.23	0.18	0.11	0.15
M208	2,6-Dimethyl-2-trans-6-octadiene	2609-23-6	Olefins	0.14	0.14	0.19	0.15	0.20	0.16	0.22	0.20	0.22	0.12	0.12	0.11	0.05	0.09
M209	Limonene	138-86-3	Olefins	2.53	2.31	2.51	2.24	2.81	2.61	2.83	2.75	2.99	0.02	2.32	1.81	1.32	2.15
M210	cis-β-Ocimene	3338-55-4	Olefins	0.15	0.18	0.22	0.19	0.24	0.20	0.22	0.21	0.02	-	0.15	0.13	0.07	0.12
M211	1,2,4,4-Tetramethylcyclopentene	65378-76-9	Olefins	-	0.01	0.02	0.02	0.02	0.02	0.09	0.01	-	0.01	0.01	0.01	0.00	0.01
M212	1,5-Heptadiene, 2,6-dimethyl-	6709-39-3	Olefins	0.01	0.01	0.01	0.01	0.01	0.01	0.01	0.01	0.01	0.00	0.00	-	-	-
M213	α-Pinene	80-56-8	Olefins	-	0.11	0.13	-	0.20	0.13	0.13	-	0.13	0.09	0.09	0.06	0.04	0.06
M214	2,6-Dimethyl-1,3,5,7-octatetraene, E,E-	460-01-5	Olefins	0.03	0.04	0.05	0.04	0.06	0.04	0.05	0.04	0.05	0.03	-	-	-	-
M215	cis-Calamenene	72937-55-4	Olefins	-	-	-	0.01	0.02	0.01	0.02	0.01	0.01	0.01	0.01	0.01	0.01	-
M216	Cyclohexene, 5-methyl-3-(1-methylethenyl)-, trans-(-)-	56816-08-1	Olefins	0.01	1.04	-	-	0.02	1.31	1.21	1.38	1.40	1.33	1.34	0.00	-	-
M217	1,3-Hexadiene, 3-ethyl-2-methyl-	61142-36-7	Olefins	-	0.01	0.01	0.02	-	-	0.02	0.01	-	-	0.02	0.01	0.02	0.02
M218	(1R,3aS,5aS,8aR)-1,3a,5a-Trimethyl-4-methylenedecahydrocyclopenta[c]pentalene	71596-72-0	Olefins	0.01	0.01	0.01	0.01	0.01	-	0.01	-	0.01	-	-	0.01	-	-
M219	1,3,8-p-Menthatriene	18368-95-1	Olefins	-	-	0.02	0.02	0.01	0.01	0.01	0.02	0.03	-	-	-	-	0.02
M220	(+)-d-Cadinene	483-76-1	Olefins	-	-	0.00	0.01	0.01	-	0.00	-	0.00	-	-	0.01	-	0.01
M221	eremophilene	10219-75-7	Olefins	0.00	-	-	-	-	0.01	0.01	0.01	0.01	0.01	-	-	-	0.01
M222	α-Muurolene	10208-80-7	Olefins	-	-	-	-	0.00	0.00	0.00	0.00	0.00	0.00	-	0.01	-	-
M223	Camphene	79-92-5	Olefins	-	-	-	-	-	-	-	0.25	0.30	-	0.22	0.17	-	0.20
M224	Longifolene	475-20-7	Olefins	-	-	-	-	-	0.01	0.01	-	0.01	-	-	-	-	0.01
M225	3,3,5,5-Tetramethylcyclopentene	38667-10-6	Olefins	0.02	-	-	-	-	-	-	-	0.01	0.04	-	-	-	-
M226	3-Tetradecene, (Z)-	41446-67-7	Olefins	0.01	-	-	0.00	-	-	-	-	-	-	0.01	-	-	-
M227	(-)-10-epizonarene	41929-05-9	Olefins	-	-	-	-	-	0.01	0.01	-	0.01	-	-	-	-	-
M228	Bicyclo[2.2.1]heptane, 2,2-dimethyl-3-methylene-, (1R)-	5794-03-6	Olefins	0.17	-	0.27	-	-	-	-	-	-	0.36	-	-	-	-
M229	(1R)-2,6,6-Trimethylbicyclo[3.1.1]hept-2-ene	7785-70-8	Olefins	0.08	-	-	0.11	-	-	-	-	0.12	-	-	-	-	-
M230	1,3-Octadiene	1002-33-1	Olefins	-	-	0.01	0.00	-	-	-	-	-	-	-	-	-	-
M231	1-Tetradecene	1120-36-1	Olefins	-	-	-	-	0.01	-	-	-	-	0.00	-	-	-	-
M232	3-Carene	13466-78-9	Olefins	-	-	-	-	-	-	-	-	-	0.00	0.00	-	-	-
M233	β-Ocimene	13877-91-3	Olefins	-	-	-	0.19	-	-	-	-	-	-	-	-	-	0.13
M234	3-Tetradecene, (E)-	41446-68-8	Olefins	-	-	-	-	-	-	-	0.01	-	-	-	0.01	-	-
M235	2-Dodecene, (E)-	7206-13-5	Olefins	-	-	-	-	-	-	-	-	-	-	0.02	0.01	-	-
M236	1-Nonene	124-11-8	Olefins	-	0.00	-	-	-	-	-	-	-	-	-	-	-	-
M237	Cyclohexene, 1-methyl-5-(1-methylethenyl)-, (R)-	1461-27-4	Olefins	-	-	-	-	-	-	-	-	0.02	-	-	-	-	-
M238	(E)-4,8-Dimethylnona-1,3,7-triene	19945-61-0	Olefins	-	-	-	0.00	-	-	-	-	-	-	-	-	-	-
M239	4-Dodecene	2030-84-4	Olefins	-	-	0.01	-	-	-	-	-	-	-	-	-	-	-
M240	cis-α-Bisabolene	29837-07-8	Olefins	-	-	-	-	-	-	-	-	-	-	-	0.00	-	-
M241	1,3-Cyclopentadiene, 5-(1-methylpropylidene)-	3141-02-4	Olefins	-	-	-	-	-	-	-	0.13	-	-	-	-	-	-
M242	4-Tetradecene, (E)-	41446-66-6	Olefins	-	-	-	-	-	-	-	-	0.01	-	-	-	-	-
M243	1,5,5-Trimethyl-6-methylene-cyclohexene	514-95-4	Olefins	-	-	-	-	-	0.01	-	-	-	-	-	-	-	-
M244	2-Carene	554-61-0	Olefins	-	-	-	-	0.03	-	-	-	-	-	-	-	-	-
M245	Cyclohexene, 4-methylene-1-(1-methylethyl)-	99-84-3	Olefins	-	-	-	-	-	-	-	-	-	-	-	0.03	-	-
M246	1,3-Cyclopentadiene, 5-(1-methylethylidene)-	2175-91-9	Olefins	-	-	-	0.05	-	-	-	-	-	-	-	-	-	-
M247	1,2,3-Trimethoxybenzene	634-36-6	Ethers	-	-	-	0.00	0.01	0.01	0.01	-	0.01	0.01	0.02	0.01	0.02	0.32

M248	Benzene,4-ethyl-1,2-dimethoxy-	5888-51-7	Ethers	-	0.00	0.01	0.00	0.01	0.02	-	-	-	-	-	0.02	0.03	-
M249	Benzene, 1,2-dimethoxy-	91-16-7	Ethers	-	-	0.01	-	0.02	0.02	0.02	0.01	0.08	0.02	0.02	0.01	0.03	0.17
M250	2,3-Dimethylanisole	2944-49-2	Ethers	-	-	-	-	-	-	-	-	-	-	-	-	-	0.08
M251	2,3-Dimethoxytoluene	4463-33-6	Ethers	-	-	-	-	-	-	-	-	-	-	-	-	-	0.05
M252	2,4-Dimethylanisole	6738-23-4	Ethers	-	-	-	-	-	-	-	-	-	-	-	-	-	0.50
M253	2,5-Dimethoxyethylbenzene	1199-08-2	Ethers	-	-	-	-	-	-	0.02	0.02	-	0.03	0.03	-	-	0.58
M254	2,6-Dimethoxytoluene	5673-7-4	Ethers	-	-	-	-	-	-	-	-	-	-	-	-	-	0.02
M255	Oxepine, 2,7-dimethyl-	1487-99-6	Ethers	0.03	0.01	0.01	0.01	0.01	-	0.01	0.01	0.01	-	0.01	0.01	-	-
M256	Naphthalene, 2-methoxy-	93-4-3	Ethers	-	-	-	-	-	-	-	-	-	-	-	-	-	0.09
M257	Benzene, 1,2,3-trimethoxy-5-methyl-	6443-69-2	Ethers	-	-	-	-	-	-	-	-	-	-	0.01	-	-	0.14
M258	3,4-Dimethoxytoluene	494-99-5	Ethers	0.00	0.01	0.01	0.01	0.02	0.01	0.03	0.01	0.04	0.02	0.04	0.02	0.05	0.75
M259	3,5-Dimethylanisole	874-63-5	Ethers	-	-	-	-	-	-	-	-	-	-	-	-	-	0.17
M260	3,5-Dimethoxytoluene	4179-19-5	Ethers	-	-	-	-	-	-	-	0.01	-	0.01	-	-	-	0.06
M261	Safrole	94-59-7	Ethers	-	-	-	-	-	0.01	0.00	-	-	-	-	-	-	-
M262	Benzofuran, 5-methoxy-6,7-dimethyl-	35355-35-2	Ethers	-	-	-	-	-	-	0.01	-	-	-	-	-	0.00	0.13
M263	Benzofuran, 7-methoxy-	7168-85-6	Ethers	-	-	-	-	-	-	-	0.01	-	0.01	0.01	-	0.01	0.14
M264	2H-Pyran, 2-ethenyltetrahydro-2,6,6-trimethyl-	7392-19-0	Ethers	0.04	0.04	0.05	0.05	0.07	0.05	0.07	0.07	0.12	0.04	0.08	0.03	0.04	0.09
M265	Anethole	104-46-1	Ethers	-	-	-	-	-	-	-	-	-	-	-	-	-	0.02
M266	2H-Pyran, tetrahydro-4-methyl-2-(2-methyl-1-propenyl)-	16409-43-1	Ethers	-	-	-	0.02	-	-	-	-	-	-	-	-	-	-
M267	1H-Pyrrole, 1-ethyl-	617-92-5	heterocyclic compounds	0.28	0.28	0.29	0.29	0.34	0.27	0.23	0.24	0.20	0.18	0.17	0.20	0.15	0.09
M268	3,6-Dimethyl-2,3,3a,4,5,7a-hexahydrobenzofuran	70786-44-6	heterocyclic compounds	0.01	0.01	0.01	0.01	0.02	0.01	0.02	0.01	0.03	0.02	0.01	0.01	0.01	0.02
M269	2,4-Dimethylfuran	3710-43-8	heterocyclic compounds	-	-	0.05	-	-	-	-	-	-	-	-	-	-	-
M270	2-n-Butyl furan	4466-24-4	heterocyclic compounds	0.04	0.03	0.04	0.03	0.04	0.03	0.05	0.03	0.05	0.02	0.03	0.02	0.01	0.02
M271	Furan, 2-ethyl-5-methyl-	1703-52-2	heterocyclic compounds	0.03	0.02	0.03	-	0.01	0.00	0.03	0.03	0.03	-	0.02	0.01	-	0.02
M272	Pyridine, 2-ethyl-	100-71-0	heterocyclic compounds	-	-	-	-	-	-	-	-	-	-	-	-	-	0.02
M273	Furan, 2-ethyl-	3208-16-0	heterocyclic compounds	-	0.28	0.33	-	0.33	-	0.27	-	-	-	0.27	0.19	0.20	0.26
M274	Ethanone, 1-(1H-pyrrol-2-yl)-	1072-83-9	heterocyclic compounds	-	-	-	-	-	-	-	-	-	-	-	0.01	-	-
M275	Ethanone, 1-(2-furanyl)-	1192-62-7	heterocyclic compounds	-	0.01	0.01	-	-	0.01	-	0.01	-	-	0.01	0.01	-	-
M276	Furan, 2-propyl-	4229-91-8	heterocyclic compounds	-	-	-	-	-	-	-	0.02	-	-	-	-	-	-
M277	2,5,5,8a-Tetramethyl-3,4,4a,5,6,8a-hexahydro-2H-chromene	72746-44-2	heterocyclic compounds	0.01	0.02	0.02	0.03	0.03	0.02	0.03	0.02	0.03	0.01	0.02	0.02	0.02	0.02
M278	2H-Pyran,3,6-dihydro-4-methyl-2-(2-methyl-1-propenyl)-	1786-08-9	heterocyclic compounds	0.02	0.02	0.03	0.03	0.03	0.03	0.04	0.03	0.05	0.02	0.02	0.01	0.02	0.03
M279	3-Methyl-2-(2-methyl-2-butenyl)-furan	15186-51-3	heterocyclic compounds	0.01	0.01	0.01	-	-	0.01	-	-	-	-	0.01	-	-	-
M280	Benzofuran, 4,7-dimethyl-	28715-26-6	heterocyclic compounds	-	-	-	-	-	-	-	-	-	0.03	-	-	-	-
M281	Dibenzofuran, 4-methyl-	7320-53-8	heterocyclic compounds	-	-	-	-	-	-	-	-	-	-	-	-	-	0.01
M282	(2R,5S)-2-Methyl-5-(prop-1-en-2-yl)-2-vinyltetrahydrofuran	54750-69-5	heterocyclic compounds	0.25	0.25	0.29	0.27	0.34	0.28	0.34	0.25	0.40	0.15	0.26	0.17	0.20	0.39
M283	trans-2-(2-Pentenyl)furan	70424-14-5	heterocyclic compounds	-	-	-	-	-	-	0.18	-	-	-	-	-	-	-
M284	(2R,5R)-2-Methyl-5-(prop-1-en-2-yl)-2-vin	54750-70-8	heterocyclic	-	0.77	-	-	-	-	-	-	-	1.27	-	-	-	0.81

	yltetrahydrofuran		compounds														
M285	2H-1-Benzopyran, 3,5,6,8a-tetrahydro-2,5,5,8a-tetramethyl-, trans-	41678-29-9	heterocyclic compounds	0.01	0.01	0.01	0.01	0.02	0.02	0.02	0.02	0.02	0.01	0.02	0.01	0.03	0.05
M286	1H-Pyrrole-2-carboxaldehyde, 1-ethyl-	2167-14-8	heterocyclic compounds	0.39	0.19	0.42	0.42	-	0.37	0.42	0.43	0.43	0.27	0.34	0.44	0.35	-
M287	1-Oxaspiro[4.5]dec-6-ene,2,6,10,10-tetramethyl-	36431-72-8	heterocyclic compounds	0.13	0.24	0.23	0.38	0.24	0.28	0.31	0.25	0.34	0.14	0.27	0.27	0.14	0.14
M288	Dibenzofuran	132-64-9	heterocyclic compounds	0.01	0.02	0.02	0.01	0.03	0.03	0.03	0.02	0.04	0.02	0.02	0.02	0.02	0.10
M289	Edulan II	41678-30-2	heterocyclic compounds	0.02	0.02	0.02	0.02	0.02	0.02	0.02	0.02	0.02	0.02	0.03	0.02	0.02	0.02
M290	1H-Indene, 2,3-dihydro-1,1,3-trimethyl-3-phenyl-	3910-35-8	heterocyclic compounds	0.01	-	-	-	0.02	0.10	0.04	0.01	0.02	0.06	0.01	0.02	-	-

- indicates that the aroma component is not detected in the sample; the unit of substance content is mg/kg.
